# 新型光敏剂3^2^-(4-甲氧基苯基)-15^2^-天冬氨酸-二氢卟吩e6的药代动力学及组织分布特征

**DOI:** 10.3724/SP.J.1123.2021.01010

**Published:** 2021-12-08

**Authors:** Liu WANG, Yi DONG, Lei CAO, Yuming SUN, Yueqing LI, Weijie ZHAO

**Affiliations:** 1.大连理工大学化工与环境生命学部化工学院, 辽宁 大连 116024; 1. College of Chemical Engineering, Faculty of Chemical, Environment and Biological Science and Technology, Dalian University of Technology, Dalian 116024, China; 2.大连理工大学化学分析测试中心, 辽宁 大连 116024; 2. Chemical Analysis and Research Center, Dalian University of Technology, Dalian 116024, China; 3.大连理工大学精细化工国家重点实验室, 辽宁 大连 116024; 3. State Key Laboratory of Fine Chemicals, Dalian University of Technology, Dalian 116024, China

**Keywords:** 高效液相色谱-紫外检测, 药代动力学, 组织分布, 光敏剂, 二氢卟吩类, 荷瘤小鼠, high performance liquid chromatography-ultraviolet (HPLC-UV) detection, pharmacokinetics, tissue distribution, photosensitizer, chlorins, tumor-bearing mice

## Abstract

二氢卟吩类衍生物3^2^-(4-甲氧基苯基)-15^2^-天冬氨酸-二氢卟吩e6(DYSP-C34)是从程海湖螺旋藻中提取并合成的新型光敏剂。研究DYSP-C34在生物体内的药代动力学及组织分布过程对光动力疗法(PDT)的有效性和安全性至关重要。该文运用高效液相色谱-紫外(HPLC-UV)检测技术,建立了大鼠血浆中DYSP-C34的检测方法。采用沉淀蛋白-液液萃取法处理血浆和组织样品,采用Unitary C_18_色谱柱(250 mm×4.6 mm, 5 μm)分离,流动相为甲醇-5 mmol/L四丁基磷酸氢铵缓冲盐溶液(70:30, v/v),流速为1.0 mL/min,进样量为20 μL,检测波长为400 nm,柱温为40 ℃。实验结果表明,大鼠血浆药物质量浓度在1~200 μg/mL范围内线性良好,判定系数(*r*^2^)为0.9941。在低、中、高(8、40、120 μg/mL)3个添加水平下的提取回收率分别为74.39%、69.71%和65.89%,日内和日间相对标准偏差(RSD)均在5%以内。运用此方法测定静脉注射DYSP-C34(16 mg/kg)后大鼠血浆中以及荷瘤小鼠组织中的药物浓度,采用DAS 2.0计算出药物半衰期*t*_1/2z_为6.98 h,药-时曲线下面积AUC_(0-∞)_为1025.01 h·mg/L,平均驻留时间MRT_(0-∞)_为9.19 h。DYSP-C34在荷瘤小鼠体内的分布结果显示,DYSP-C34可以在肿瘤组织中蓄积,并具有一定的滞留作用。综上,该文建立了大鼠血浆中DYSP-C34的HPLC-UV测定方法,并进行了方法学验证,此方法简便、快速,结果准确。阐明了DYSP-C34在静脉给药方式下大鼠体内药代动力学和荷瘤小鼠组织中的分布特征,对临床合理用药和药效学研究具有重要意义。

## Abstract (en)

Photodynamic therapy (PDT) has garnered immense research interest. PDT can directly kill the cells via a combination of photosensitizer, light, and molecular oxygen. It has emerged as a promising therapeutic option for cancer treatment owing to its advantages such as minimized systemic toxicity, minimal invasiveness, high therapeutic efficacy, and potential for developing antitumor immunity. The novel photosensitizer 3^2^-(4-methoxyphenyl)-15^2^-aspartyl-chlorin e6 (DYSP-C34) was synthesized by introducing a 3^2^-aryl substitution and amino acid substituent of the Chenghai chlorin (CHC). Briefly, 3^2^-(4-methoxyphenyl) substitution was achieved via olefin metathesis reactions. The aspartic acid side chain was introduced regioselectively at C-15^2^, followed by hydrolysis to yield the target DYSP-C34. CHC with the same chemical structure as chlorin e6 was prepared from chlorophyll a, which was extracted from Spirulina powders derived from Chenghai Lake in the Yunnan province of China. This strategy successfully endowed the resultant photosensitizer with better cellular permeability and increased water solubility. In addition, the photodynamic antitumor effects of PDT largely depend on the dose of photosensitizer used, time between photosensitizer administration and light exposure, and possibly other still poorly known variables. Determination of optimal conditions for PDT requires a coordinated interdisciplinary effort. Therefore, the pharmacokinetics and tissue distribution of DYSP-C34 in vivo are critical for the efficacy and safety of PDT. Herein, a high performance liquid chromatography-ultraviolet (HPLC-UV) detection method was established for the determination of the new photosensitizer DYSP-C34 in rat plasma. The sample preparation involved a protein-precipitation and liquid-liquid extraction method. Methanol was used to precipitate proteins and chloroform was used to extract chlorins. Then, DYSP-C34 was separated on a Unitary C_18_ column (250 mm×4.6 mm, 5 μm) with a mobile phase comprising methanol and 5 mmol/L tetrabutylammonium phosphate buffer solution (70:30, v/v). The flow rate was 1.0 mL/min with UV detection using a wavelength of 400 nm at 40 ℃. Results showed that DYSP-C34 and chlorin e6 trimethyl ester (IS) were well separated under these conditions. The method was sensitive and sufficiently precise with a good linear relationship (determination coefficient (*r*^2^)=0.9941) over the range of 1-200 μg/mL in rat plasma. At three spiked levels (8, 40, and 120 μg/mL), the average recoveries were 74.39%, 69.71%, and 65.89%, respectively. The intra-day and inter-day relative standard deviations (RSDs) were lower than 5%. The precision met the requirements of biological sample determination. Furthermore, DYSP-C34 was stable in rat plasma under various storage conditions at room temperature, three freeze-thaw cycles, and long-term cryopreservation. The validated method was successfully applied to the pharmacokinetic study of DYSP-C34 after intravenous injection of a single dose in rat plasma. The pharmacokinetic parameters after intravenous injection of DYSP-C34 (16 mg/kg) were calculated. The plasma half-life (*t*_1/2z_) was 6.98 h, the area under the plasma concentration-time curve AUC_(0-∞)_ was 1025.01 h·mg/L and the mean retention time MRT_(0-∞)_ was 9.19 h. In addition, the results of DYSP-C34 distribution in tumor-bearing mice showed that DYSP-C34 could accumulate in tumor tissues, with higher concentrations in liver and kidney tissues, and lower concentrations in heart, spleen, and lung tissues. In summary, a specific, simple, and accurate HPLC-UV method was developed and validated for the determination of DYSP-C34 in rat plasma and tumor-bearing mouse tissues. The pharmacokinetics of DYSP-C34 after intravenous administration in rats and the tissue distribution characteristics of tumor-bearing mice were clarified for the first time. It is significant for clinical rational drug use and pharmacodynamic research. Therefore, choosing an appropriate time for light treatment time can achieve the best photodynamic effect. The results of pharmacokinetics and tissue distribution of DYSP-C34 provide vital guidance for subsequent pharmacodynamic research and further clinical trials in terms of dosage, light time, light toxicity and side effects.

光动力疗法(PDT)是一种基于光敏剂的非侵入性肿瘤治疗方法^[1]^。PDT可以有效地和选择性地破坏肿瘤组织而不损伤周围健康组织,因此获得了越来越多的关注^[2,3]^。二氢卟吩类光敏剂是目前研究较多的肿瘤光动力治疗药物,主要包括二氢卟吩e6(chlorin e6)及其衍生物^[4,5,6]^。此类光敏剂具有组分单一、结构明确、肿瘤摄入率高、光动力作用强和毒性低等优点^[7]^。

本课题组前期^[8]^成功从云南程海湖螺旋藻粉中提取并制备了程海二氢卟吩(Chenghai chlorin, CHC,结构同二氢卟吩e6),同时对CHC进行结构修饰,通过烯烃复分解反应引入甲氧基苯基^[9]^,使用碳二亚胺类缩合剂(EDCI)区域选择性地引入天冬氨酸侧链,合成了具有两亲性的二氢卟吩衍生物3^2^-(4-甲氧基苯基)-15^2^-天冬氨酸-二氢卟吩e6,编号为DYSP-C34(结构见图1)。除上述二氢卟吩类光敏剂的优点外,DYSP-C34还弥补了大多数光敏剂水溶性较差的缺陷,适合研制应用于临床注射的水针剂。另外,光敏剂在肿瘤组织蓄积和滞留的能力直接影响光动力疗法杀伤肿瘤的效果。因此,DYSP-C34的药代动力学及肿瘤组织分布特性研究对其临床合理用药和药效学方面具有重要的指导意义。

**图1:**
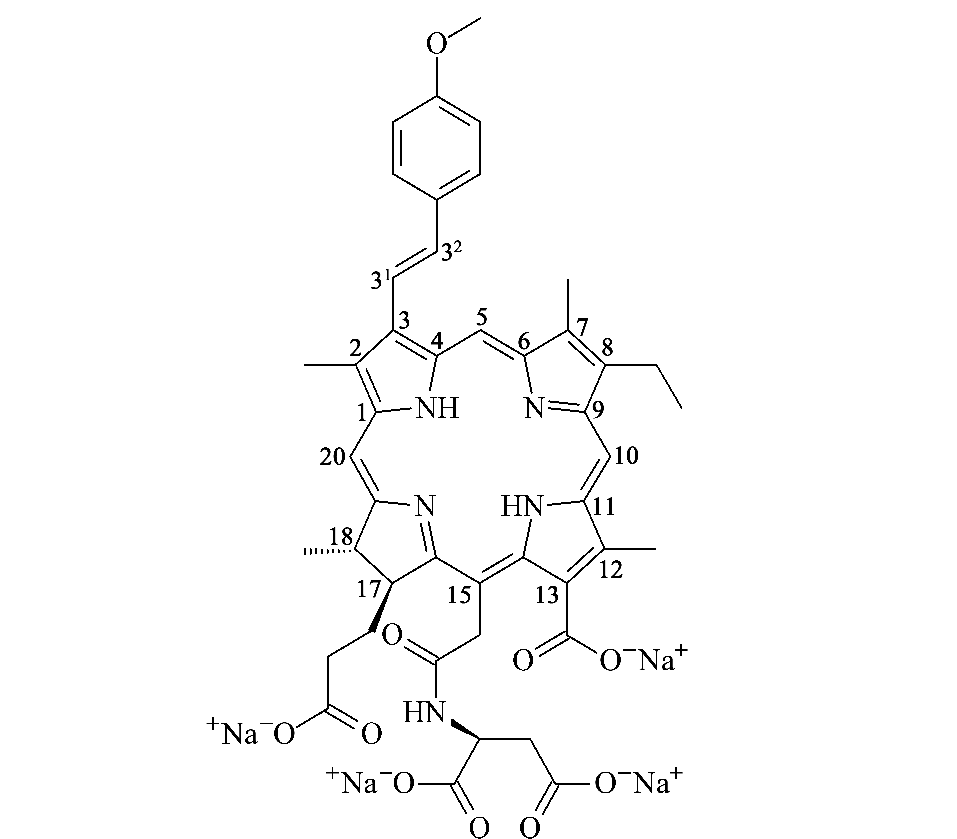
DYSP-C34的结构式

本实验将采用HPLC-UV方法定量测定大鼠血浆中的DYSP-C34,并评价方法的特异性、准确性、精密度和稳定性,探究DYSP-C34在大鼠体内的药代动力学及荷瘤小鼠组织中的分布情况。

## 1 实验部分

### 1.1 仪器与试剂

Waters e2695高效液相色谱仪、2489UV/Vis紫外检测器(美国Waters公司); BT224S电子天平(北京赛多利斯天平有限公司); MG-2200氮气吹扫仪(东京理化株式会社); D1008小型离心机(大龙兴创实验仪器(北京)有限公司); FSH-2A高速可调均质机(常州金坛良友仪器有限公司); Milli-Q型超纯水机(美国Millipore公司)。

光敏剂DYSP-C34为实验室自合成,纯度为98.48%。内标二氢卟吩e6三甲酯(CHCTME)为实验室自合成,结构见附图1(详见http://www.chrom-China.com),纯度为96.64%。甲醇(美国Fisher Scientific公司)为色谱纯;二氯甲烷(天津市进丰化工有限公司);四丁基磷酸氢铵(纯度98%,安耐吉化学股份有限公司,批号:DX5REU4Q)为分析纯;二甲基亚砜(DMSO,美国Amresco公司)为生物纯;0.9%氯化钠注射液(辽宁民康制药有限公司,批号:A190510B-2);肝素钠(大连美仑生物技术有限公司)。

Sprague Dawley(SD)大鼠,雄性,无特定病原体(SPF),体重220~250 g; Institute of Cancer Research(ICR)小鼠,雄性,周龄为6~8周,SPF级,体重18~25 g,由本溪长生生物股份有限公司提供,生产许可证号:SCXK(辽)2015-0001。

本实验采用小鼠肝癌细胞(hepatoma-22, H22,由国家实验细胞资源共享平台提供)构建小鼠皮下异位移植瘤模型。使用含有10%血清(PAN^TM^ SERATECH No. ST30-3302)、含1%青霉素的链霉素溶液(HyClone, No. SV30010)的RPMI 1640(Gibco, No. C11875500BT)培养基将细胞在37 ℃、5%CO_2_的培养箱中悬浮培养。

### 1.2 标准溶液配制

DYSP-C34标准溶液:称取DYSP-C34 40.0 mg,加入适量生理盐水溶解,在25 mL棕色容量瓶中用生理盐水定容,制得1.6 mg/mL标准储备液,置于-20 ℃冰箱避光保存,备用。

CHCTME标准溶液:称取CHCTME 10.0 mg,加入适量DMSO溶解,在10 mL棕色容量瓶中用DMSO定容,制得1.0 mg/mL标准储备液,置于-20 ℃冰箱避光保存,备用。

### 1.3 色谱条件

色谱柱:Unitary C_18_柱(250 mm×4.6 mm, 5 μm,华普科仪(大连)科技有限公司);柱温:40 ℃;流动相:(A)甲醇和(B)5 mmol/L四丁基磷酸氢铵缓冲盐溶液;流速:1 mL/min。梯度洗脱程序:0~10 min, 70%A~95%A; 10~15 min, 95%A~100%A; 15~20 min, 100%A。进样量:20 μL;检测波长:400 nm。

### 1.4 造模及给药

1.4.1 大鼠给药

选取6只健康雄性SD大鼠,称重,按照16 mg/kg的给药剂量尾静脉注射DYSP-C34标准溶液,大鼠在给药前禁食12 h,自由饮水。

1.4.2 荷瘤小鼠造模及给药

将H22小鼠肝癌细胞(3×10^5^个/只)接种于ICR小鼠右侧背部皮下,正常饲养并密切观察ICR小鼠的健康状况。待肿瘤体积生长到100~150 mm^3^(一般情况下为接种后8 d左右),可用作组织分布研究。荷瘤小鼠称重后,按照16 mg/kg的给药剂量尾静脉注射DYSP-C34标准溶液,给药前禁食12 h,自由饮水。

### 1.5 生物样品收集与处理

1.5.1 血浆样品

大鼠给药后,分别于5、15、30 min以及1、1.5、2、4、6、12、24 h自大鼠眼后静脉丛取血0.2 mL,置于含有肝素钠的离心管内,以8000 r/min离心5 min后取上层血浆,于-80 ℃冰箱冷冻保存,分析前,于室温下解冻。

取0.1 mL解冻后的血浆,置于2 mL离心管中,依次加入0.1 mL生理盐水、5 μL CHCTME标准溶液、200 μL 0.1 mol/L磷酸、0.5 mL甲醇和1 mL二氯甲烷溶液,充分振荡,以8000 r/min离心5 min,取下层清液于2 mL离心管中,氮气吹扫(50 ℃)至无液体剩余,加入1 mL初始流动相(甲醇-四丁基磷酸氢铵(7∶3, v/v))溶液复溶,0.45 μm滤膜过滤,然后进行HPLC分析。

1.5.2 组织样品

在给药后1、2、4、6、8、12 h脱颈处死小鼠,取心脏、肝脏、脾脏、肺、肾脏以及肿瘤组织,置于生理盐水中清洗,滤纸擦干后称重。加入4倍体积的生理盐水,剪碎组织匀浆后取100 μL匀浆液,置于2 mL离心管中。按1.5.1节方法沉淀蛋白质及提取药物。

## 2 结果与讨论

### 2.1 色谱条件优化

二氢卟吩衍生物是一类强荧光物质,已有许多报道采用荧光分析方法检测生物样品中的二氢卟吩类光敏剂^[10,11]^。但环境因素可能会对其荧光光谱的位置和强度产生强烈影响,荧光效率也易受温度、溶剂和酸度影响^[12]^。DYSP-C34是由3个吡咯环和1个还原的吡咯环组成的具有共轭*π*键体系的大杂环分子,这些吡咯环通过4个亚甲基键连接。由于强共轭体系,DYSP-C34在400 nm和660 nm的波长处具有很强的紫外吸收,使得直接的紫外检测法具有足够的敏感性^[12,13]^。与660 nm相比,DYSP-C34在400 nm的吸收更强,故将检测波长设置为400 nm。

二氢卟吩类光敏剂DYSP-C34的合成路线见附图1,核磁、质谱数据见附图2~4。由附图1可以发现,通过结构修饰后的DYSP-C34含有多个羧基,极性较大,并具有一定酸性。选择常规流动相体系甲醇-水或乙腈-水时,DYSP-C34在C_18_柱上没有保留。为了获得适当的保留时间,将离子对试剂(四丁基磷酸氢铵)加入到流动相中,DYSP-C34的羧基离子与离子对试剂离子形成中性的离子对化合物,在非极性固定相中溶解度增大,从而使其分离效果改善,并确定最优色谱条件(见1.3节)。在此条件下,空白和加标大鼠血浆样品的色谱图见图2。可以看出,样品和内标经色谱分离后,DYSP-C34的保留时间为8.0 min左右,内标的保留时间为12.2 min左右,且不受血浆中内源性物质的干扰,峰形良好,并得到很好的分离。

**图2:**
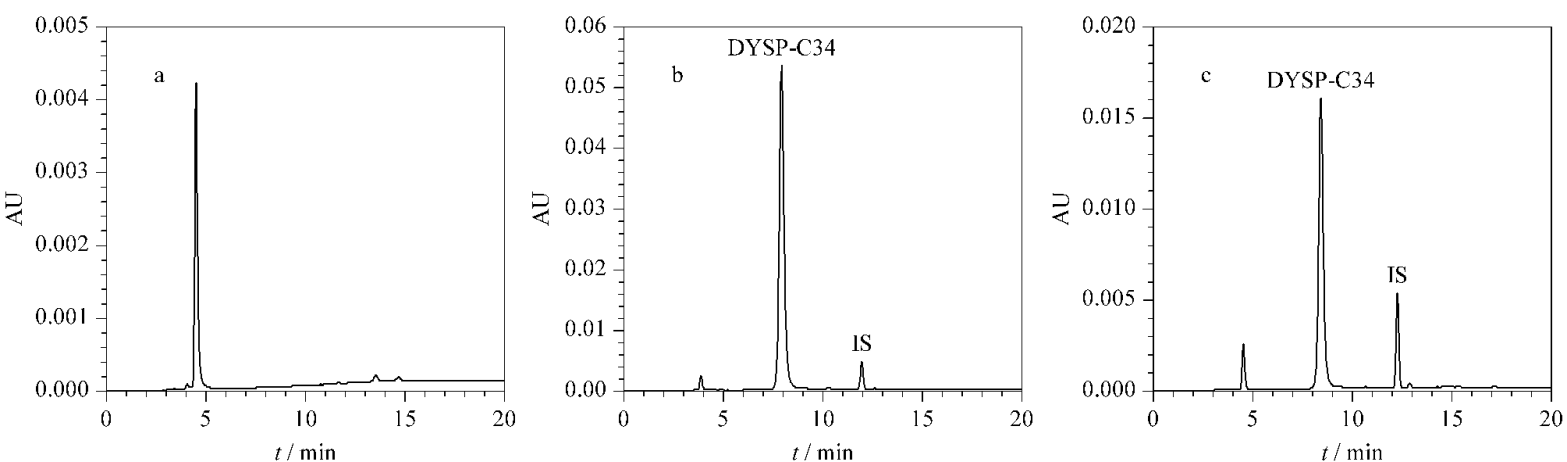
(a)空白大鼠血浆样品、(b)空白大鼠血浆加标准品(120 μg/mL)和(c)尾静脉注射DYSP-C34(16 mg/kg)2 h后大鼠血浆样品的色谱图

### 2.2 方法学考察

2.2.1 线性关系

取空白大鼠血浆0.1 mL,加入0.1 mL不同浓度的DYSP-C34,使样品中DYSP-C34的质量浓度分别为1、4、8、16、40、80、120和200 μg/mL。按上述1.5.1节所述方法进行样品处理和HPLC测定。采用加权最小二乘法计算标准曲线方程,以DYSP-C34与内标的峰面积比值为纵坐标(*y*), DYSP-C34的质量浓度为横坐标(*x*, μg/mL),得到的回归方程为*y*=0.1010*x*+0.1398,判定系数(*r*^2^)为0.9941。

采用同样方法测定并计算DYSP-C34在荷瘤小鼠组织中的线性回归方程。结果见附表1,不同组织中的DYSP-C34在1~200 μg/mL范围内呈良好的线性关系,*r*^2^均大于0.99。

2.2.2 回收率与精密度

提取回收率为空白血浆中加入药物经提取后的药物响应值与空白血浆处理后加入相同量药物响应值的比值。取大鼠空白血浆0.1 mL,加入0.1 mL不同含量的DYSP-C34,使药物的质量浓度分别为8、40、120 μg/mL;用空白血浆提取液分别配制8、40、120 μg/mL的DYSP-C34药物溶液,分别对上述溶液进行分析。如表1所示,大鼠血浆在高、中、低3个水平下的提取回收率为65.89%~74.39%, RSD≤3.61%,说明该方法可靠。

**表1:** DYSP-C34在大鼠血浆中3个水平下的提取回收率(*n*=3)

Analyte	Added/(μg/mL)	Recovery/%	RSD/%
DYSP-C34	120	65.89	3.61
	40	69.71	0.32
	8	74.39	1.52

在大鼠空白血浆中加入DYSP-C34标准品溶液,配制成8、40、120 μg/mL的样品溶液,按上述1.5.1节方法处理样品并进行HPLC测定。同一天内测定6次,连续测定3 d,分别考察日内和日间精密度。实验结果见表2,该方法的日间和日内精密度均在5%以内,符合2015版《中华人民共和国药典》对于生物样品分析的要求^[14]^。以上结果表明该方法重复性良好,可应用于大鼠血浆中DYSP-C34的测定。

**表2:** DYSP-C34在大鼠血浆中的日内及日间精密度(*n*=6)

Analyte	Added/ (μg/mL)	Inter-day				Intra-day		
		Found/ (μg/mL)	RSD/ %			Found/ (μg/mL)		RSD/ %
DYSP-C34	120	124.04	3.57		127.28		2.43	
	40	40.19	3.49		40.16		4.40	
	8	8.68	1.20		7.90		1.11	

2.2.3 稳定性

取大鼠空白血浆0.1 mL,加入0.1 mL不同浓度的DYSP-C34,使药物的质量浓度分别为8、40、120 μg/mL,将样品室温放置(2 h)、反复冻融3次或者放入-80 ℃冰箱冷冻保存30 d后,按上述1.5.1节所述方法处理样品并进行HPLC测定,考察DYSP-C34在大鼠血浆中的稳定性。实验结果显示,高、中、低3个水平的血浆样品在上述条件下测定的精密度均在10%以内,表明DYSP-C34血浆样品在储存及测定过程中均是稳定的。

### 2.3 DYSP-C34在大鼠体内的药代动力学

采用药代动力学计算软件DAS 2.0^[15]^,按照非室模型进行拟合并计算药代动力学参数。在大鼠中单次尾静脉注射DYSP-C34(16 mg/kg)后,DYSP-C34的平均血药浓度随时间变化曲线如图3所示,主要药代动力学参数见附表2。DYSP-C34尾静脉注射后的半衰期*t*_1/2z_为6.984 h,药-时曲线下面积AUC_(0-∞)_为1025.01 h·mg/L,平均驻留时间MRT_(0-∞)_为9.19 h。药物在大鼠血浆中的半衰期较长,表明药物在血浆中清除较慢,可以维持稳定的血药浓度,有利于延长体内循环时间,这对临床给药方案具有一定的指导意义。因此,在临床应用中,可以通过单次静脉给药的方式来施用DYSP-C34。

**图 3:**
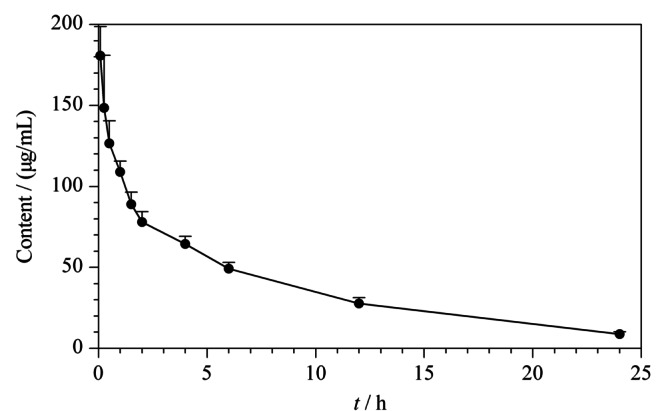
大鼠单次尾静脉注射16 mg/kg DYSP-C34后的平均血药浓度-时间曲线(*n*=6)

### 2.4 DYSP-C34在荷瘤小鼠体内的组织分布

将收集的小鼠组织按照1.5.2节处理并进行HPLC分析,根据DYSP-C34在荷瘤小鼠组织中的线性回归方程(见附表1)计算得到DYSP-C34在荷瘤小鼠组织中浓度随时间变化曲线,如图4所示。结果表明,给药后1 h, DYSP-C34在小鼠心、肝、脾、肺、肾以及肿瘤组织中均有分布。随着时间的增加,DYSP-C34在肝脏组织中的浓度均高于其他组织,提示其主要通过肝脏代谢;其次,肾脏中DYSP-C34达峰时浓度高于心脏、脾和肺,提示肾脏可能为DYSP-C34的主要排泄器官。

**图4:**
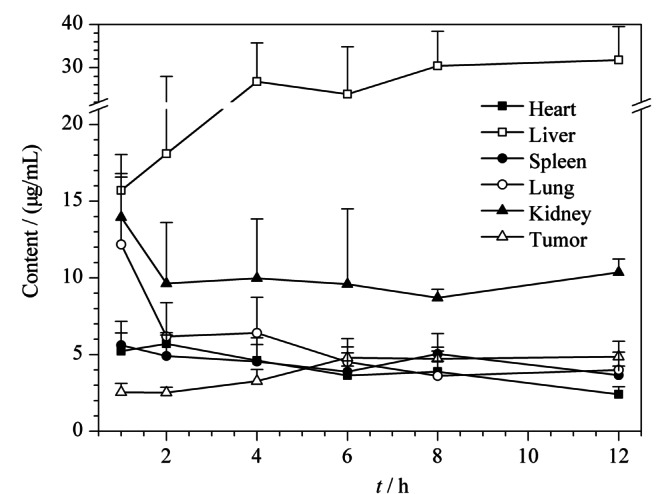
荷瘤小鼠单次尾静脉注射16 mg/kg DYSP-C34后组织中的药物浓度随时间变化曲线(*n*=5)

肿瘤组织内的DYSP-C34含量在给药后总体呈现出随时间延长而上升的趋势,在6~12 h后,肿瘤组织中DYSP-C34含量变化不十分明显,维持在一个相对稳定的水平。DYSP-C34可以在肿瘤组织中蓄积,使其作用时间延长。在6 h时,心脏、脾和肺的DYSP-C34浓度小于肿瘤组织,因此可以选择该时间点作为异位移植瘤小鼠的光动力光照处理时间。需要注意的是,DYSP-C34在肝脏和肾脏组织从2 h之后一直处于较高浓度,所以DYSP-C34介导的光动力疗法用于肝癌和肾癌的治疗时一定要注意其导致的副作用。

## 3 结论

建立并验证了一种简便、快速、准确的HPLC-UV方法测定大鼠血浆中新型二氢卟吩光敏剂DYSP-C34,成功测定了大鼠尾静脉注射16 mg/kg DYSP-C34后的血药浓度,并运用此方法检测DYSP-C34在荷瘤小鼠不同组织样品中的浓度。结果表明,DYSP-C34是一种体内循环时间长、肿瘤组织蓄积性好、在体内部分组织代谢速率快的光敏剂。单次静脉注射给药后,DYSP-C34在大鼠体内的血药浓度和荷瘤小鼠组织中的药物浓度变化结果,为后续的药效学研究和临床使用提供了用药剂量、光照时间、光毒副作用等方面的参考和依据。
